# Radiographic Evaluation and Changes in Bone Density of the Humeral Side after Reverse Total Shoulder Arthroplasty

**DOI:** 10.3390/jcm12247698

**Published:** 2023-12-15

**Authors:** Daisuke Soma, Toru Ichiseki, Shusuke Ueda, Masaru Sakurai, Norio Kawahara

**Affiliations:** 1 Department of Orthopaedic Surgery, Kanazawa Medical University, Daigaku 1-1, Uchinada-machi, Kahoku-gun 920-0293, Ishikawa, Japan; ds0924@kanazawa-med.ac.jp (D.S.);; 2 Social and Environmental Medicine, Kanazawa Medical University, Daigaku 1-1, Uchinada-machi, Kahoku-gun 920-0293, Ishikawa, Japan; m-sakura@kanazawa-med.ac.jp

**Keywords:** bone mineral density, bone resorption, cuff tear arthroplasty, osteoporosis, rotator cuff tear, reverse total shoulder arthroplasty

## Abstract

After artificial joint surgery, bone density may decrease around the artificial joint; thus, postoperative bone density evaluation around the artificial joint is crucial. We investigated changes in bone mineral density and performed radiographic evaluation around the stem after reverse shoulder arthroplasty (RSA) surgery in 17 males (18 shoulders) and 19 females (19 shoulders), aged >65 years, with >1-year follow-up. In total, 20 and 17 cases involved massive rotator cuff tears and rotator cuff tear arthropathy, respectively. The Comprehensive Reverse Shoulder System (Standard Ingrowth) was used for all cases and cement was used in eight patients due to bone fragility. We examined lucent lines, loosening, bone resorption, and spot welds in non-cemented cases using plain radiography and postoperative bone density changes around the stem using dual-energy X-ray absorptiometry (DEXA). Lucent lines and bone resorption occurred in 5 (13.5%) and 19 (51.4%) shoulders, respectively. No loosening occurred. Compared to stem bone density at 2 weeks postoperatively, the decrease rate was the largest in the proximal medial humerus. One-year postoperative bone density was not related to sex, age, cement use, or preoperative diagnosis. Higher preoperative bone density was better maintained postoperatively. Furthermore, 1 year post RSA, spot welds were observed in approximately 48.2% of cases at the distal medial portion of the stem coating, and bone resorption occurred in the proximal medial humerus in 43.2% of cases. Therefore, postoperative bone density is related to preoperative bone density, suggesting the importance of maintaining high preoperative bone density.

## 1. Introduction

Reverse shoulder arthroplasty (RSA) provides stability by extending the deltoid muscle, moving the center of rotation forward and downward, and pressuring the center of rotation. This procedure is an effective treatment for shoulder joint pain and increasing the range of motion. RSA reportedly has a relatively good 5-year survival rate of 91% when considering revision surgery as the endpoint. However, approximately 21% of RSA revision surgeries are reportedly caused by aseptic loosening of the humerus or periprosthetic fracture [1]. Therefore, performing postoperative plain radiographic evaluation of the humeral side is important. Several studies have investigated bone resorption on the humeral side after RSA. Inoue et al. reported that periprosthetic bone resorption after RSA most commonly affects the proximal part of the humerus, such as the greater tuberosity, the lateral diaphysis, and the proximal medial portion of the humerus [2]. Rotator cuff tears may contribute to osteoporosis in the proximal humerus [3,4].

There is a concern that decreased bone density around artificial joints increases the risk of periprosthetic fractures and loosening [5,6,7,8,9]. Additionally, deterioration of bone quality and bone loss on the humeral side have been identified as major challenges during revision surgery. Therefore, evaluating bone resorption in simple radiographs and following up on bone density are important. For example, in the hip and knee joints, in addition to radiographic evaluation after artificial joint replacement, some reports have focused on bone density [10,11,12,13,14,15,16]. Reports on decreases in bone density around an artificial joint, particularly after total hip arthroplasty (THA), have been widely seen. Inoue et al. reported that the decrease in preoperative bone density is related to fractures of the greater trochanter during the perioperative period [17]. Additionally, Liu et al. reported that the female patients older than 50 years lost significantly more bone density than the male patients [18]. Changes in bone density after THA have also been reported to be more pronounced in individuals with sarcopenia associated with aging than in patients without sarcopenia [19]. Therefore, the decrease in bone density around the artificial joint is considered a noteworthy event in the postoperative course. In Japan, RSA is indicated for individuals aged 65 years and older. Given that bone density tends to be decreased in older adults even before surgery, changes in bone density during the postoperative course are considered particularly important in this population. However, there have been no reports of changes in postoperative bone density around the stem after RSA.

Therefore, in this study, in addition to a plain radiographic evaluation in the short term after RSA, we investigated bone density by using dual-energy X-ray absorptiometry (DEXA) and studied factors involved in bone density status at 1 year postoperatively in patients who underwent RSA.

## 2. Materials and Methods

### 2.1. Patients

This study included 36 patients (37 shoulders) who had undergone RSA at our hospital from April 2017 to July 2022 and whose bone mineral densities were measured preoperatively, as well as at 2 weeks, 6 months, and 1 year after surgery. We excluded cases with no revision on the stem side during the follow-up period, cases with glenoid fracture leading to revision, cases with rheumatoid arthritis, and cases in which the patient was bedridden due to severe complications. This study was conducted in accordance with the Declaration of Helsinki and approved by the Institutional Ethics Committee of Kanazawa Medical University (protocol code I575 and date of approval 23 December 2020).

Fifteen patients had hypertension, eight patients had heart disease, and five patients had diabetes. In these patients, the comorbid conditions were well controlled, and no patients had poorly controlled disease or severe complications that could affect bone density. The average body mass index was 23.22 (min: 18.66, max: 29.22, standard deviation 2.523) kg/m^2^. The average age at surgery was 73.5 years (65–86 years). In total, 17 patients were males (18 shoulders) and 19 were females (19 shoulders). Preoperative diagnoses included 20 cases of massive rotator cuff tears and 17 cases of rotator cuff tear arthropathy. In total, 25 shoulders involved the dominant side and 12 involved the non-dominant side. None of the cases examined in this study received continuous treatment for osteoporosis before or after surgery.

The stem used in all cases was an on-lay type Comprehensive Reverse Shoulder System (Zimmer Biomet, Warsaw, IN, USA). This model has an angle of 135 degrees and a coating of approximately 40 mm from the proximal part of the stem to the distal part, and the shape and stem length are classified as a standard. Trabecular Metal Reverse Plus (Zimmer Biomet) was used on the glenoid side in all cases. In addition, in eight cases wherein bone fragility was observed during surgery, cement fixation was performed to increase stem fixation.

### 2.2. Evaluation Items

#### 2.2.1. Radiographic Evaluation

**Figure 1:**
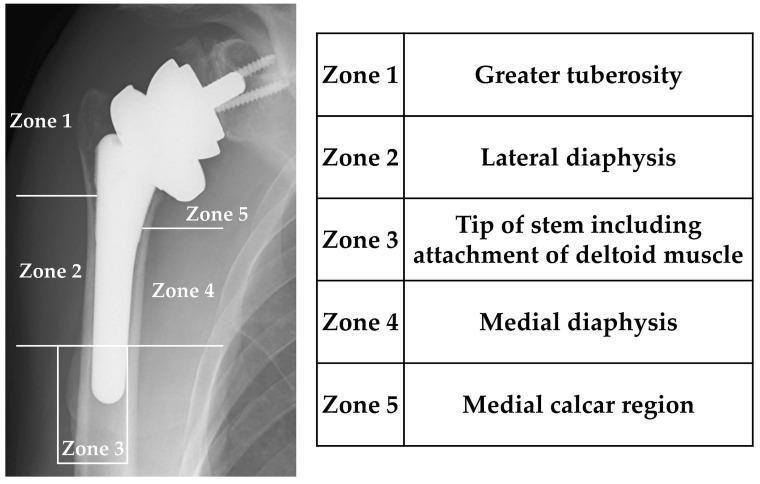
Locations of bone resorption.

We investigated the frequency and location of bone resorption, lucent lines, loosening and spot welds in non-cemented cases using plain radiography. Previous reports indicate that stress shielding is more common in the proximal part of the stem and less common in the mid-to-distal part. Therefore, in this study, we modified the zone classification [2] of Inoue et al. and classified them from Zones 1 to 5 centered on the proximal part of the humerus. The greater tuberosity, the lateral shaft of the humerus, the distal portion of the stem including the deltoid attachment, the medial shaft of the humerus, and the proximal medial (calcar) of the humerus were Zones 1–5, respectively (Figure 1). Bone resorption was defined as Grades 0–4 according to the report by Inoue et al. [2] and surveyed for each zone. Lucent lines were defined as clear lines around the stem by comparing plain radiographs taken 2 weeks after surgery and again at the final observation. Following the report of Melis et al. [20], implant loosening was defined as displacement of the humeral component between the time of the initial post-operative radiograph and the most recent follow-up, or if radiolucency ≥2 mm was present in more than three zones. Similarly, in cementless cases, we evaluated spot welds, which indicate adhesion between the stem and the bone. All imaging assessments were evaluated by three shoulder surgeons.

#### 2.2.2. Postoperative Change in Bone Density around the Stem

Bone density was calculated using the DEXA method. The measurement sites for bone density around the stem were Zones 1–5, the same as in the radiographic evaluation. The bone mineral density measured at 2 weeks after surgery for each zone was used as a control, and the following examinations were performed.

##### Change Rate at 1 Year after Surgery

The rate of change in bone density at 1 year after surgery was calculated as (Bone density at 1 year after surgery—Bone density at 2 weeks after surgery [control]/Bone density at 2 weeks after surgery × 100 [%]). The rate of change in bone density was compared in each zone.

##### Factors Contributing to Bone Density in Each Zone at 1 Year Postoperatively

Multiple regression analysis was used to examine the significance of age, sex, presence or absence of cement use, preoperative diagnosis, and preoperative bone density in affecting bone density in each zone at 1 year postoperatively.

### 2.3. Statistical Analysis

One-way analysis of variance was used to compare the rate of bone density change in each zone at 1 year after surgery, with Bonferroni’s test as post hoc test. Multiple regression analysis was used to test for factors involved in bone density at 1 year postoperatively in each zone. All data analysis was performed using IBM SPSS Statistics software (version 28; IBM Corp., Armonk, NY, USA), and *p* < 0.05 was considered statistically significant.

## 3. Results

### 3.1. Evaluation at 1 Year after Surgery Using Plain Radiography

**Figure 2:**
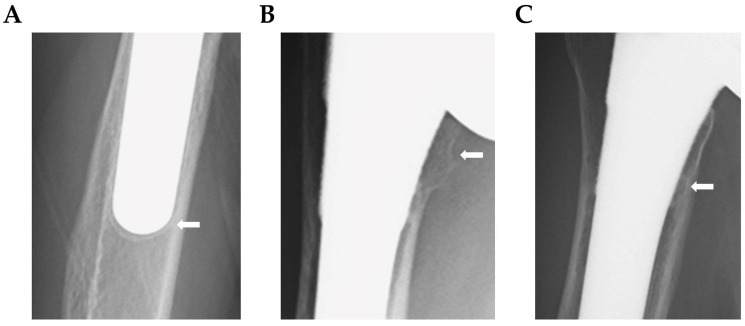
Representation of imaging findings. (**A**) Lucent line. (**B**) Bone resorption (Grade 4). (**C**) Spot welds.

Lucent lines within 2 mm were observed in five shoulders (13.5%), which all occurred in Zone 3 (Figure 2A). Bone resorption was observed in 19 shoulders (51.4%), including 5 (13.5%), 6 (16.2%), and 16 (43.2%) in Zones 1, 2, and 5, respectively. Grade 4 bone resorption was observed in only three shoulders (8.1%), in Zone 5, while resorption up to Grade 2 was seen in the other cases (Figure 2B). In total, 29 cases did not require cement use, 14 (48.2%) of which demonstrated spot welds, all in Zone 4. Six cases (20.7%) had spot welds in Zone 2 (Figure 2C). All spot welds appeared in the distal part of the stem coating. No loosening was observed in any of the cases (Table 1).

### 3.2. Bone Density Changes around the Stem

#### 3.2.1. Rate of Change in Bone Density in Each Zone at 1 Year after Surgery, Compared to 2 Weeks after Surgery

Table 2 shows the rate of bone density change in each zone at 1 year after surgery relative to 2 weeks. A slight increase in the rate of change in bone density was observed in Zone 4, while the rate of change was decreased in other zones. When comparing the rates of change in each zone, Zone 5 had the lowest rate (approximately 22%), showing a significant decrease as compared with Zones 1, 2, and 4 (Table 2).

#### 3.2.2. Factors Associated with Bone Density at 1 Year after Surgery

Sex, age, presence or absence of cement use, preoperative diagnosis, and preoperative bone density were considered factors that could affect postoperative bone density and were included in multiple regression analysis. Preoperative bone density was identified as an associated factor in all zones, except for Zone 2. In other words, the higher the preoperative bone density, the better the postoperative bone density could be maintained. Sex was a significant factor in 1-year postoperative bone density in Zone 3. Conversely, age, presence or absence of cement use, and preoperative bone density were not significant factors influencing bone density in any of the zones (Table 3).

## 4. Discussion

In cases requiring revision after RSA, aseptic loosening and fractures around the stem side of the humerus have been reported to be more frequent than loosening and fractures on the scapular side [1,21]; therefore, considering the stem area is crucial. Hence, in this study, we examined bone density, which is considered a risk factor for postoperative fractures, in addition to performing radiographic assessment of the stem side after RSA surgery. We found lucent lines and bone resorption, particularly at the medial proximal humerus, in 13.5% and 51.4% of shoulders, respectively. In total, 14 of 29 cementless cases showed spot welds at the distal medial portion of the stem coating. The decrease rate in bone density was greatest in the proximal medial humerus. One-year postoperative bone density was not related to age, cement use, or preoperative diagnosis. Higher preoperative bone density was maintained better postoperatively.

Several studies have reported on the incidence of lucent lines, bone resorption, and implant loosening around the stem after RSA. For example, Grey et al. reported a short-term incidence of lucent lines of 11.9%. The incidence of lucent lines was 15.9% and 9.5% for cemented and cementless stems, respectively [22]. We found a similar incidence of lucent lines (13.5%). Conversely, Melis et al. reported that lucent lines were observed in 57% of patients at an average postoperative time of 9.6 years [20], suggesting the need for caution in the long-term course. Furthermore, spot welds in non-cemented cases appeared predominantly in the distal part of the coating of the stem in Zones 2 and 4, similar to the locations of spot welds observed after THA [23,24,25]. Bone resorption reportedly occurs around the humeral stem relatively early in the postoperative period, with most sites appearing in the proximal part of the stem [2,26,27]. In this study, bone resorption was found in 13.5% and 43.2% of cases in Zones 1 and 5, respectively, while three shoulders (8.1%) showed Grade 4 (complete loss of cortical bone) in Zone 5 in the proximal part of the stem.

When bone resorption progressed in the greater tuberosity after RSA, the deltoid wrapping effect was weakened due to the decreased volume of the proximal lateral humerus. Since it causes relative loosening of the deltoid muscle, we believe that it may lead to decreased shoulder joint range of motion and muscle strength and may cause instability. In this study, bone resorption in the greater tuberosity of Zone 1 reached Grade 2. However, further caution is required, since these data were found in 13.5% of the cases within the short period of 1 year.

Currently, no reports on bone density around the stem after RSA are available. Periprosthetic bone density loss after arthroplasty is believed to increase the risk of requiring revision surgery [5,6]. Therefore, considering bone mineral density is important. Hence, we examined changes in bone density around the stem during the postoperative course of RSA using the DEXA method. We showed that bone mineral density decreased in all zones except for Zone 4 (proximal medial diaphysis of the humerus), even in the short period of 1 year after surgery. In Zone 5, the rate of occurrence of bone resorption was particularly high, and the rate of decrease in bone density was the highest, at approximately 22%. This may be because this site is less subject to mechanical loading and is more proximal than the sites of spot welds in non-cemented cases. Similarly, in the hip joint, bone density decreases in the proximal medial area [9,10], and bone resorption reportedly occurs more proximally to the site of spot welds [28]. In this study, we consider that this was because the site was subjected to less loading because it was proximal to Zone 4, where many spot welds appeared. Bone density generally decreases with age, and mechanical loading is important to prevent this. We considered changes in bone density, taking into account the mechanical load on the stem side after RSA. Zone 5, which exhibited a notable decrease in bone density in our study, is proximal to Zone 4, where numerous spot welds appeared in our patients. This suggests that the site experiences less mechanical loading, which is considered as one of the contributing factors to its significant decrease in bone density. Similar findings have been reported at the hip joint, where bone density decreases in the proximal medial shaft of the stem [9,10], and bone resorption occurs closer to the stem than spot weld sites do [28]. Furthermore, the absence of muscle attachments in Zone 5 is considered to be another reason for the lower bone density in this less mechanically loaded area.

In contrast, for Zone 1, since the deltoid wrapping characteristic of RSA is a site where mechanical load is applied, it is possible that the decrease in bone density was only approximately 5% in the short term. However, bone resorption in Zone 1 occurred in approximately 13.5% of cases. In non-cemented cases, it was more proximal than the appearance of spot welds. Similar to Zone 5, sufficient monitoring in this region is therefore important. In this study, bone resorption at this site reached Grade 2; however, this may worsen over the long term. There are concerns about the weakness of the deltoid muscle that is required for deltoid wrapping and the decrease in muscle strength due to aging. Hence, if a stem with rigidity that differs from that of bone is inserted, it is possible that bone density will decrease, and that bone resorption will occur in the proximal region of the stem within a short period of time. Therefore, we believe that follow-up evaluation with plain radiographs, bone density measurements, and other approaches is important. On the other hand, in this study, we found a slight change in bone density in Zones 2, 3, and 4. Zone 3 includes the deltoid attachment. Therefore, considering the characteristic use of the deltoid muscle to move the arm after RSA, sufficient mechanical loading may be supplied by muscular forces. Additionally, the observed significant difference between the sexes only in Zone 3 may be attributed to the stronger muscle strength in males. Since muscle strength measurements were not conducted in this study, further investigation of this matter is needed. Bone density changes in Zones 2 and 4 may have been underestimated due to the common occurrence of spot welds in these areas. The propensity for spot welds in these regions, where the fixation between the stem and bone is more likely to occur, may indicate that they are subjected to greater mechanical loading. Further investigation, including stress analysis, will be necessary to understand this aspect. Our data indicate that, when inserting a stem with a different rigidity from that of bone, reduction in bone density and increased bone resorption in the proximal part of the stem may occur within a short period, emphasizing the need for thorough observation. Therefore, in future, not only plain radiography but also evaluations of bone density will be important. Moreover, while this study focused on postoperative bone density changes; future studies should consider evaluating bone quality in addition to bone density in image evaluation.

We also examined factors related to postoperative bone density and concluded that preoperative bone density is related to postoperative bone density, suggesting that it is important to maintain high preoperative bone density. Previous reports on bone mineral density after THA have revealed that decreased bone mineral density increases the risk of periprosthetic fractures and the likelihood of requiring revision surgery [5,6]. Since this study focused on RSA in older adults aged ≥65 years, aggressive therapeutic intervention for osteoporosis that can improve and maintain bone density before surgery is required to prevent postoperative bone density loss. Similarly, some studies [3,4] have reported decreased bone density in the greater tuberosity of Zone 1, particularly due to rotator cuff tears. Therefore, monitoring changes in bone density before surgery in patients with rotator cuff tears requiring RSA is crucial. Postoperative administration of teriparatide and denosumab is reportedly effective in maintaining bone quality around artificial joints [29,30,31,32]. Hence, we strongly recommend that osteoporosis drugs should be considered after surgery. In future, in addition to following bone density changes after RSA surgery in the long term, examining the presence or absence of intervention for osteoporosis before and after surgery and the effect of the type of device used (Onlay or Inlay) is important. It has also been reported that the filling rate of the humeral stem is related to stress shielding when a cementless stem is used [33]. In this study, we did not examine the filling rate of humeral stem because we examined cemented and cementless cases. In the future, it will be necessary to study the filling rate of the humeral stem by increasing the number of cases and the follow-up period of non-cemented stems.

Furthermore, as a future perspective, when postoperative periprosthetic fractures occur or significant osteolysis becomes evident, it will be important to utilize advanced imaging technologies, such as three-dimensional computed tomography, as reported by Moldovan et al. [34] on intra-articular fractures of the knee joint. This will facilitate understanding of the morphology of the fracture and of the osteolytic part and will allow simulation of proper reduction of the fracture site in places with reduced bone density. We also believe that it is necessary to evaluate the relationship between the incidence of such events and bone density.

Moreover, this study revealed that inserting a stem with a different rigidity from that of bone in RSA will cause a bone density loss of approximately 22%, depending on the site, within the short period of 1 year. Since there is a concern that bone density may further decrease over time, continuous, careful follow-up is considered vital.

There were several limitations in our study. First, this study focused on the same model, namely the standard stem, and it remains unclear whether different lengths or shapes of stems would exhibit similar changes in bone density. Second, the artificial joints used in this study were all of the onlay type, and bone density assessment for the inlay type was not conducted. Therefore, it is unclear whether similar trends would be observed with all types of artificial joints. Third, this study focused on older adults aged 65 years and above, and the bone density changes following RSA in younger individuals are unknown.

## 5. Conclusions

In this study, bone resorption occurred within a short period after RSA in older adult patients aged ≥65 years. The rate of decrease in bone density was greatest in the proximal medial portion of the stem in Zone 5. Preoperative bone density was significantly related to bone density at 1 year postoperatively, suggesting the importance of preoperative intervention for osteoporosis. Bone loss around the artificial shoulder joint is involved in the long-term durability of the prosthesis and is a challenge during revision when loosening of the implant or periprosthetic fracture develops. Therefore, evaluating bone density around the artificial joint and actively intervening in osteoporosis treatment is crucial.

## Figures and Tables

**Table 1:** Frequency of bone resorption, lucent line, spot welds, and loosening in each zone.

	Zone 1	Zone 2	Zone 3	Zone 4	Zone 5	Total
Bone resorption	5 (13.5%)	6 (16.2%)	0	0	16 (43.2%)	19/37 (51.4%)
Lucent line	0	0	5 (13.5%)	0	0	5/37 (13.5%)
Spot welds	0	6 (20.7%)	0	14 (48.2%)	0	14/29 (48.2%)
Loosening	0	0	0	0	0	0/37

**Table 2:** Measured values and rate of change in bone mineral density in each zone at 2 weeks and 1 year after in each zone.

DEXA	2 Weeks after Surgery	1 Year after Surgery	Rate of Change (%) ^1^
Zone 1	0.619 ± 0.164	0.565 ± 0.133	−5.308 ± 23.349
Zone 2	0.914 ± 0.206	0.860 ± 0.188	−4.820 ± 20.461
Zone 3	0.932 ± 0.205	0.879 ± 0.164	−6.190 ± 12.794
Zone 4	0.843 ± 0.174	0.901 ± 0.190	7.860 ± 16.285
Zone 5	0.698 ± 0.182	0.521 ± 0.258	−22.626 ± 34.326

^1^ Rate of change is calculated as [1 year postoperatively − 2 weeks postoperatively (control)/2 weeks postoperatively × 100].

**Table 3:** Results of multivariate regression analysis to identify factors influencing bone mineral density in each zone at 1 year after surgery.

	*p* Value	β (95% CI)
Zone 1		
Sex (male vs. female)	0.547	−0.032 (−0.671 to 0.926)
Age	0.621	0.002 (−0.007 to 0.012)
Cemented vs. non-cemented	0.320	−0.059 (−0.178 to 0.060)
Preoperative diagnosis	0.317	−0.047 (−0.141 to 0.047)
Preoperative DEXA	0.016 *	0.480 (0.095 to 0.866)
Zone 2		
Sex (male vs. female)	0.169	0.101 (−0.046 to 0.249)
Age	0.749	0.002 (−0.011 to 0.015)
Cemented vs. non-cemented	0.416	−0.056 (−0.193 to 0.082)
Preoperative diagnosis	0.127	−0.087 (−0.199 to 0.026)
Preoperative DEXA	0.078	0.269 (−0.032 to 0.570)
Zone 3		
Sex (male vs. female)	0.013 *	0.110 (0.025 to 0.196)
Age	0.346	0.004 (−0.005 to 0.012)
Cemented vs. non-cemented	0.347	−0.042 (0.132 to 0.048)
Preoperative diagnosis	0.130	−0.058 (−0.135 to 0.018)
Preoperative DEXA	<0.001 *	0.503 (0.297 to 0.709)
Zone 4		
Sex (male vs. female)	0.055	0.134 (−0.003 to 0.270)
Age	0.910	0.001 (−0.012 to 0.014)
Cemented vs. non-cemented	0.624	0.032 (−0.100 to 0.165)
Preoperative diagnosis	0.619	−0.026 (−0.130 to 0.079)
Preoperative DEXA	0.047 *	0.443 (0.007 to 0.879)
Zone 5		
Sex (male vs. female)	0.679	0.043 (−0.168 to 0.254)
Age	0.399	−0.008 (−0.027 to 0.011)
Cemented vs. non-cemented	0.601	−0.059 (−0.285 to 0.168)
Preoperative diagnosis	0.266	−0.106 (−0.296 to 0.085)
Preoperative DEXA	0.018 *	0.808 (0.147 to 1.469)

CI; confidence interval, * *p* < 0.05.

## Data Availability

Data are contained within the article.
